# The dyslipidemia-associated SNP on the *APOA1*/*C3*/*A5* gene cluster predicts post-surgery poor outcome in Taiwanese breast cancer patients: a 10-year follow-up study

**DOI:** 10.1186/1471-2407-13-330

**Published:** 2013-07-05

**Authors:** Mei-Chi Hsu, Kuo-Ting Lee, Wei-Chiang Hsiao, Chih-Hsing Wu, Hung-Yu Sun, I-Ling Lin, Kung-Chia Young

**Affiliations:** 1 Research Center for Medical Laboratory Biotechnology, College of Medicine, National Cheng Kung University, Tainan, Taiwan; 2 Department of Medical Laboratory Science and Biotechnology, College of Medicine, National Cheng Kung University, Tainan, Taiwan; 3 Department of Surgery, College of Medicine, National Cheng Kung University, Tainan, Taiwan; 4 Department of Surgery, Yang Ming Hospital, Chiayi, Taiwan; 5 Department of Family Medicine, College of Medicine, National Cheng Kung University, Tainan, Taiwan; 6 Department of Medical Laboratory Science and Biotechnology, College of Health Sciences, Kaohsiung Medical University, Kaohsiung, Taiwan; 7 Center of Infectious Disease and Signaling Research, College of Medicine, National Cheng Kung University, Tainan, Taiwan

**Keywords:** *APOA1*/*C3*/*A5*, SNP, *APOA1* rs670, Lymph node micrometastasis, Post-surgery, Prognosis

## Abstract

**Background:**

Post-surgery therapies are given to early-stage breast cancer patients due to the possibility of residual micrometastasis, and optimized by clincopathological parameters such as tumor stage, and hormone receptor/lymph node status. However, current efficacy of post-surgery therapies is unsatisfactory, and may be varied according to unidentified patient genetic factors. Increases of breast cancer occurrence and recurrence have been associated with dyslipidemia, which can attribute to other known risk factors of breast cancer including obesity, diabetes and metabolic syndrome. Thus we reasoned that dyslipidemia-associated nucleotide polymorphisms (SNPs) on the *APOA1*/*C3*/*A5* gene cluster may predict breast cancer risk and tumor progression.

**Methods:**

We analyzed the distribution of 5 selected *APOA1*/*C3*/*A5* SNPs in recruited Taiwanese breast cancer patients (n=223) and healthy controls (n=162). The association of SNP (*APOA1* rs670) showing correlation with breast cancer with baseline and follow-up parameters was further examined.

**Results:**

*APOA1* rs670 A allele carriage was higher in breast cancer patients than controls (59.64% *vs*. 48.77%, p=0.038). The rs670 A allele carrying patients showed less favorable baseline phenotype with positive lymph nodes (G/A: OR=3.32, 95% CI=1.77-6.20, p<0.001; A/A: OR=2.58, 95% CI=1.05-6.32, p=0.039) and negative hormone receptor expression (A/A: OR=4.85, 95%CI=1.83-12.83, p=0.001) in comparison to G/G carriers. Moreover, rs670 A/A carrying patients had higher risks in both tumor recurrence (HR=3.12, 95% CI=1.29-7.56, p=0.012) and mortality (HR=4.36, 95% CI=1.52-12.47, p=0.006) than patients with no A alleles after adjustments for associated baseline parameters. Furthermore, the prognostic effect of rs670 A/A carriage was most evident in lymph node-negative patients, conferring to the highest risks of recurrence (HR=4.98, 95% CI=1.40-17.70, p=0.013) and mortality (HR=9.87, 95%CI=1.60-60.81, p=0.014) than patients with no A alleles.

**Conclusions:**

*APOA1* rs670 A/A carriage showed poor post-surgery prognosis in Taiwanese lymph node-negative breast cancer patients, whose prognosis were considered better and adjuvant treatment might be less stringent according to currently available assessment protocols. Our findings suggest that *APOA1* rs670 indicate a post-surgery risk of breast cancer disease progression, and that carriers of this SNP may benefit from more advanced disease monitoring and therapy regimens than the current regular standards. Furthermore, control of lipid homeostasis might protect *APOA1* rs670 minor allele carriers from breast cancer occurrence and progression.

## Background

Breast cancer is currently the leading cause of cancer deaths in females worldwide, and also the second most common cancer after lung cancer [1]. The current therapeutic regimens for operable early-stage breast cancers include endocrine therapy, chemotherapy and radiotherapy. The assessment tools for post-surgery planning are currently based on clinicopathological evaluations, but are unsatisfactory requiring finer adjustments [2]. The risk factors for breast cancer poor prognosis include positive sentinel lymph node metastasis, hormone receptor negativity, larger tumor size, younger age, and menopausal status [2]. In contrast to the Western population, the breast cancer incidence peaks at a younger age in Oriental Asians, which include Taiwanese [3,4]. Nevertheless, the westernized dietary pattern and lifestyle in Taiwan in the last two decades has increased the incidences of metabolic disorders, including dyslipidemia, as well as breast cancer in Taiwanese females [4].

Both cohort and case–control epidemiological observations have demonstrated the association of metabolic syndrome, obesity and diabetes with increased breast cancer risk [5,6]. Dyslipidemia often occur in parallel to obesity and diabetes, and is a component of metabolic syndrome [7]. Dyslipidemia in the forms of hypertriglyceridemia, hypercholesterolemia, and low high density lipoprotein-cholesterol (HDL-C) have been observed in breast cancer patients of several ethnic groups [8-11]. Moreover, in large cohort studies dyslipidemia is associated with increased breast cancer risk and poor prognosis [12-14]. The intake of lipid-lowering drugs or agents in women was associated with reduced breast cancer occurrence and recurrence risk [15-17]. Consistently, mammary tumor growth and metastasis was accelerated in a hyperlipidemic murine model [18].

The *APOA1*/*C3*/*A5* gene cluster transcribes for apolipoproteins (apo) A1, C3 and A5, which regulate HDL-formation and lipoprotein lipase activity [19]. Single-nucleotide polymorphisms (SNPs) on the *APOA1*/*C3*/*A5* gene cluster are associated with metabolic syndrome, dyslipidemia and diabetes [19]. *APOA1* rs670 is associated with altered HDL-C levels, and increased risks of coronary artery disease and metabolic syndrome [20-25]. *APOC3* rs2854116 and rs2854117 are correlated to insulin resistance and non-alcoholic liver steatosis [26]. *APOA5* rs662799 and rs2075291 carriers are known to have increased plasma triglyceride [27,28], and our recent study showed correlation of *APOA5* rs662799 with central obesity in males [29]. Though the effects of these SNPs on *APOA1*/*C3*/*A5* on metabolic disorders have been widely studied and reviewed, their contributions to breast cancer have not been determined in detail.

In this study, we first tested the correlation of 5 selected well-known SNPs on the *APOA1*/*C3*/*A5* gene cluster with breast cancer in a case–control manner. Furthermore, we analyzed the effect of the SNPs associated with greatest breast cancer risk on patient baseline tumor characteristics, and their post-surgery outcome after a mean follow-up period of 10.4 years. Finally, we specifically examined the prognostic value of *APOA1* rs670 in lymph node-negative breast cancer patients.

## Methods

### Patient recruitment

Taiwanese female breast cancer patients (n=223, 48.4±10.2 years, ranged 29–75 years) received surgical intervention plus axillary/sentinel lymph node dissection during 1999–2005 at National Cheng Kung University Hospital (NCKUH) and Tainan Hospital, and were followed-up to November 2012. Healthy female controls (n=162, 43.0 ± 8.8 years, ranged 19.0–69.0 years.) were also recruited. This is a continuation study of Hsiao et al., 2004 [30]. Bodyweight (kg) and body height (m) were measured at the time of mastectomy, and used for calculating body mass index (BMI, kg m^-2^). The diagnosis was confirmed by histological examinations of mammary and node specimens. Estrogen receptor (ER) and progesteron receptor (PR) expressions in primary breast tumor were determined as described previously [31]. Individuals with at least one first-degree or second-degree female relatives affected by breast cancer were considered to have a family history. This information was obtained by interview with patients and their family members. The recruited patients received tamoxifen (TAM) (n=92), TAM and chemotherapy (n=56), TAM and radiotherapy (n=10), and triple-therapy (n=55). This study received approval from the local institutional review board (NCKUH IRB) and signed informed consent was obtained from the patients.

### Genomic DNA extraction, SNP genotyping and linkage analysis

Genomic DNA was extracted from white blood cells using the Puregene DNA Isolation Kit (Gentra Systems, Minneapolis, MN, USA). According to criteria described in Hsu *et al*. [29], we selected five SNPs on the *APOA1*/*C3*/*A4*/*A5* gene cluster that follows: *APOA1* rs670, *APOC3* rs2854116, *APOC3* rs2854117, *APOA5* rs662799 and *APOA5* rs2075291. The SNP genotypes were determined using commercial real-time PCR primer and probes from Applied Biosystems (ABI, Foster City, CA, USA) (*APOA5* rs662799 and *APOA5* rs2075291) and TIB MOBIOL (Berlin, Germany) (*APOA1* rs670, *APOC3* rs2854116 and *APOC3* rs2854117). Fluorescence data from real-time PCRs were collected by a Step-One-Plus Sequence Detection System (ABI) or LightCycler 480 (Roche, St. Louis, MO, USA). Haploview [32] was used for the analysis of SNP linkage disequilibrium, and Hardy-Weinberg equilibrium, and haplotype analysis. SNP linkage disequilibrium test results with logarithm of odds (LOD) socores ≥2 and pair-wise D’>0.80 were considered as significant linkage.

### Statistical analysis

The association of SNP with breast cancer risk, baseline clinical parameters and post-surgery progression was analyzed by Chi-squared test. The odds ratio for unfavorable baseline characteristics and events in post-surgery progression was analyzed by binary or multi-nominal logistic analysis. The differences in BMI, age, and mean years in survival were analyzed by one-way ANOVA. The survival curves of *APOA1* rs670 genotype carriers were plotted by Kaplan-Meier analysis. The hazard ratio for overall and recurrence-free survival was calculated by Cox proportional-hazards regression analysis. Possible confounders including unfavorable baseline characteristics, age and BMI were adjusted for in regression analysis. Statistical analysis was performed using SPSS 13 (SPSS Inc., Chicago, DE, USA). In all cases, p-values ≤0.05 were considered statistically significant.

## Results

### Demographic characteristics of recruited breast cancer patients

The baseline characteristics of the recruited breast cancer patients are shown in Table 1. The mean BMI of breast cancer patients at baseline was 23.36±3.79 kg m^-2^. The major tumor type of recruited patients was infiltrating ductal carcinoma (86.55%), and the tumor occurrence side was evenly distributed (right breast: 44.84%, left breast: 51.12%, bilateral: 4.04%). The recruited patients were mainly of early-stage breast cancer, as 71.30% of patients had tumors <5 cm, 69.06% were of tumor stages 0–2, and 64.57% of patients had single or double positive for ER and PR. Additionally, 53.81% of patients had detectable lymph node involvement, and only 5.83% had a family history of breast cancer. The follow-up period of the breast cancer patients ranged from 0.14 to 24.52 years (median: 9.93 years), mounting to 2261.68 person-years in total. About three-forth of patients (73.99%) remained progression-free of recurrence or death. The mean years for recurrence-free and overall survival were 9.26±5.31 and 10.14±5.11, respectively (Table 1).

**Table 1:** **Demographic data of recruited breast cancer patients** (**n**=**223**)

**Parameters**	**Values**
Baseline	
Age (years)	48.4±10.2 (29–75)
BMI^a^ (kg m^-2^)	23.36±3.79 (15.06-38.05)
Tumor type	
Infiltrating ductal carcinoma	193 (86.55%)
Ductal carcinoma *in situ*	17 (7.62%)
Others	11 (4.93%)
Unknown	2 (0.90%)
Side	
Right	100 (44.84%)
Left	114 (51.12%)
Both	9 (4.04%)
Tumor size	
<5cm	159 (71.30%)
≥5cm	26 (11.66%)
Unknown	38 (17.04%)
Lymph node involvement	
Positive	121 (53.81%)
Negative	90 (40.36%)
Unknown	12 (5.83%)
TMN staging	
Stages 0-2	154 (69.06%)
Stages 3-4	30 (13.45%)
Unknown	39 (17.49%)
ER/PR status	
Single or double positive	144 (64.57%)
Negative	52 (23.32%)
Unknown	27 (12.11%)
Family history	
Positive	13 (5.83%)
Negative	203 (91.03%)
Unknown	7 (3.14%)
Post-surgery follow-up	
Therapies	
TAM only	92 (41.26%)
TAM and chemotherapy	56 (25.11%)
TAM and radiotherapy	10 (4.48%)
Triple therapy	55 (24.66%)
Unknown	10 (4.48%)
Recurrence	
With	56 (25.11%)
Without	167 (74.89%)
Mortality	
With	33 (14.80%)
Without	190 (85.20%)
Progression-free	165 (73.99%)
Mean survival years	
Recurrence-free	9.26±5.31 (0.04-24.52)
Overall	10.14±5.11 (0.14-24.52)

NOTE: Values shown are mean±s.d., or number of patients (% of n).

^a^Data from 185 patients. Bold type indicates *p*<0.050.

Abbreviations: *BMI* Body mass index, *ER* estrogen receptor, *PR* progesterone receptor, *TAM* tamoxifen.

### *ApoA1* rs670 was associated with increased breast cancer risk

The distributions of the tested SNPs (*APOA1* rs670, *APOC3* rs2854116, *APOC3* rs2854117, *APOA5* rs662799 and *APOA5* rs2075291) fitted the Hardy-Weinberg equilibrium in both breast cancer patients and healthy controls (Additional file 1). Different linkage patterns of the tested SNPs were observed between breast cancer patients (Additional file 2). Significant linkage (LOD≥ 2, D’> 0.80) was observed among *APOA1* rs670, *APOC3* rs2854116 and *APOC3* rs2854117 (D’=0.84-0.88), and between *APOA5* rs662799 and *APOA5* rs2075291 in healthy controls (D’=0.86). In contrast, the linkage among *APOA1* rs670, *APOC3* rs2854116 and *APOC3* rs2854117 was lost in breast cancer patients, while significant linkage was only observed between the two *APOA5* SNPs (D’=1.00).

The frequency of *APOA1* rs670 A allele (G/A + A/A) carriers in breast cancer patients was significantly higher than that in healthy controls (59.64% *vs*. 48.77%, *p*=0.038, Additional file 3). On the other hand, the genotype and allele frequencies of all other SNPs were comparable between breast cancer patients and healthy controls. The post-surgery therapies received by the different *APOA1* rs670 genotype carriers were comparable (*p*=0.151). Therefore we tested the contribution of *APOA1* rs670 to breast cancer in recruited patients cross-sectionally at baseline and longitudinally at follow-up.

### *APOA1* rs670 was associated with sentinel lymph node-positivity and tumor ER/PR negativity at baseline

Upon analysis of *APOA1* rs670 against breast tumor clinical parameters, we found the mean age and BMI of different *APOA1* rs670 genotype carriers were comparable at baseline (Table 2). In contrast, *APOA1* rs670 was associated with nodal involvement as well as tumor hormone receptor expression (Table 2 and 3). Baseline lymph node positivity was more frequent in patients carrying *APOA1* rs670 G/A or A/A genotypes than in those carrying G/G genotype (G/A: 51.89%, A/A: 48.15%, G/G: 24.44%, *p*=0.002) (Table 2). Moreover, *APOA1* rs670 G/A or A/A carriage also significantly increased the frequency of having tumors double negative for ER/PR expression than G/G carriage in patients (G/A: 24.53%, A/A: 48.15%, G/G: 14.44%, *p*=0.006) (Table 2). In contrast, *APOA1* rs670 was not associated with tumor occurrence side, type, size, and stage or a family history (Table 2). The odds of being lymph node-positive in *APOA1* rs670 G/A carrying patients (OR=3.32, 95% CI=1.77-6.20, *p*< 0.001) and *APOA1* rs670 A/A carriers (OR=2.58, 95% CI=1.05-6.32, *p*=0.039) was higher than G/G carriers (Table 3). On the other hand, *APOA1* rs670 A/A carriers have significantly higher odds in having ER/PR double-negative tumors than their G/G counterparts (OR=4.85, 95% CI=1.83-12.83, *p*=0.001) (Table 3).

**Table 2:** ***APOA1 *rs670 A allele carriage was associated with lymph node and hormone receptor status at baseline**

	**G/G (n=90)**	**G/A (n=106)**	**A/A (n=27)**	***P *value**^**a**^
Age	46.61±10.14 (29.25-74.55)	49.98±10.24 (29.87-74.57)	47.83±9.81 (29.25-67.91)	0.067
BMI^b^	23.47±4.14 (15.06-34.48)	23.03±4.16 (2.25-38.05)	23.77±3.83 (18.18-32.05)	0.654
Tumor type				
Infiltrating ductal carcinoma	73 (81.11%)	97 (91.51%)	23 (85.19%)	0.332
Ductal carcinoma *in situ*	10 (11.11%)	5 (4.72%)	2 (7.41%)	
Others	5 (5.56%)	4 (3.77%)	2 (7.41%)	
Unknown	2 (2.22%)	0 (0.00%)	0 (0.00%)	
Side				
Right	35 (38.89%)	52 (49.06%)	13 (48.15%)	0.704
Left	51 (56.67%)	50 (47.17%)	13 (48.15%)	
Bilateral	4 (4.44%)	4 (3.77%)	1 (3.70%)	
Tumor size				
<5cm	64 (71.11%)	76 (71.70%)	19 (70.37%)	0.227
≥5cm	6 (6.67%)	16 (15.09%)	4 (14.81%)	
Unknown	20 (22.22%)	14 (13.21%)	4 (14.81%)	
Lymph node involvement				
Positive	22 (24.44%)	55 (51.89%)	13 (48.15%)	**0**.**002**
Negative	61 (67.78%)	46 (43.40%)	14 (51.85%)	
Unknown	7 (7.78%)	5 (4.72%)	0 (0.00%)	
TMN staging				
Stages 0-2	63 (70.00%)	71 (66.98%)	20 (74.07%)	0.194
Stages >2	7 (7.78%)	19 (17.92%)	4 (14.81%)	
Unknown	20 (22.22%)	16 (15.09%)	3 (11.11%)	
ER/PR status				
Single or double positive	63 (70.00%)	68 (64.15%))	13 (48.15%)	**0**.**006**
Negative	13 (14.44%)	26 (24.53%)	13 (48.15%)	
Unknown	14 (15.56%)	12 (11.32%)	1 (3.70%)	
Family history				
Positive	4 (4.44%)	3 (2.83%)	(0.00%)	0.823
Negative	81 (90.00%)	97 (91.51%)	25 (92.59%)	
Unknown	5 (5.56%)	6 (5.66%)	2 (7.41%)	

NOTE: Data shown represent number of patients (% of n) or mean±s.d.. Bold type indicates *p*<0.050.

^a^, results of Chi-squared analysis or ANOVA test, depending on the type of parameter analyzed.

^b^, results from 185 patients.

Abbreviations: *ER* estrogen receptor, *PR* progesterone receptor.

**Table 3:** ***APOA1 *rs670 A allele carrying patients had increased odds of lymph node involvement and hormone receptor expression negativity at baseline**

	**Multinominal logistic regression (using *APOA1 *rs670 G/G as reference)**					
	***APOA1 *rs670 G/A**			***APOA1 *rs670 A/A**		
	**OR**	**95% Cl**	**P value**	**OR**	**95% Cl**	**P value**
Tumor type (using infiltrating ductal carcinoma as reference)						
Ductal carcinoma *in situ*	0.38	0.12-1.15	0.086	0.64	0.13-3.11	0.575
Others	0.6	0.16-2.32	0.461	1.27	0.23-6.99	0.784
Unknown	0	0-0	N/A	0	0-0	N/A
Side (using right as reference)						
Left	0.66	0.37-1.18	0.160	0.69	0.28-1.66	0.402
Bilateral	0.67	0.16-2.87	0.593	0.67	0.07-6.59	0.734
Tumor size (using <5cm as reference)						
≥5cm	2.25	0.83-6.08	0.111	2.25	0.57-8.79	0.245
Unknown	0.59	0.28-1.26	0.173	0.67	0.21-2.21	0.515
Lymph node involvement (using positive as reference)						
Negative	3.32	1.77-6.20	<**0**.**001**	2.58	1.05-6.32	**0**.**039**
Unknown	0.95	0.28-3.18	0.930	0	0-0	N/A
TMN staging (using stages 0–2 as reference)						
Stages >2	2.41	0.95-6.11	0.064	1.8	0.48-6.79	0.385
Unknown	0.71	0.34-1.49	0.364	0.47	0.13-1.76	0.263
ER/PR status (using positive as reference)						
Negative	1.85	0.88-3.92	0.106	4.85	1.83-12.83	**0**.**001**
Unknown	0.79	0.34-1.85	0.592	0.35	0.04-2.87	0.326
Family history (using negative as reference)						
Positive	0.63	0.14-2.88	0.548	0	0-0	N/A
Unknown	1	0.30-3.40	0.997	1.3	0.24-7.09	0.765

NOTE: Bold type indicates *p*<0.050.

Abbreviations: *CI* confidence interval, *ER* estrogen receptor, *PR* progesterone receptor, *OR* odds ratio.

### *APOA1* rs670 A allele carriage was associated with poor post-surgery outcomes

Higher proportions of *APOA1* rs670 G/A and A/A carrying breast cancer patients developed recurrence or death than G/G carriers (Table 4). The higher incidences of recurrence in *APOA1* rs670 G/A and A/A carriers translate higher risks to 2.07-fold (95% CI=1.03-4.14, *p*=0.041) and 3.44-fold (95% CI=1.33-8.86, *p*=0.011) risk as compared with G/G carriers, respectively (Table 5). However, the increase in risk of recurrence in *APOA1* rs670 G/A carriers may be confounded by their increased odds in worse baseline phenotype. After adjustment for lymph node status, the statistical significance remained in A/A carriers (OR=2.83, 95% CI, 1.05-7.61, *p*=0.040), while that in G/A carriers were diminished (*p*=0.238) (Table 5). The risk of death was significantly increased in *APOA1* rs670 A/A carriers (OR=6.03, 95% CI=2.08-17.51, *p*<0.001) and this persisted after adjustments for baseline confounders of lymph node (*p*=0.007) and ER/PR (*p*=0.010) status, while no significant risk was observed in G/A carriers with or without adjustments (Table 5). The mean years in recurrence-free survival and overall survival of *APOA1* rs670 A/A carriers were significantly shorter than that of G/A and G/G counterparts (all *p*<0.001) (Table 4). Despite the significance in increased recurrence risk, the *APOA1* rs670 G/A breast cancer carriers had mean survival years comparable to their G/G counterparts.

**Table 4:** ***APOA1 *rs670 A allele carriage was associated with poor post**-**surgery outcomes**

	**G/G (n=90)**	**G/A (n=106)**	**A/A (n=27)**	***P *value**^**a**^
Recurrence	15 (16.67%)	30 (28.30%)	11 (40.74%)	**0**.**021**
Mortality	8 (8.89%)	15 (14.15%)	10 (37.04%)	**0**.**002**
Mean survival years				
Recurrence-free	9.63±4.97 (0.16-21.38)	10.03±5.45 (0.04-24.52)	5.36±4.11 (0.18-17.21)	<**0**.**001**
Overall	10.30±4.67 (0.16-21.38)	10.98±5.32 (0.14-24.52)	6.33±4.00 (0.55-17.21)	<**0**.**001**

NOTE: Data shown represent number of patients (% of n) or mean±s.d.. Bold type indicates *p*<0.050.

^a^, results of Chi-squared analysis or ANOVA test, depending on the type of parameter analyzed.

Abbreviations: *ER* estrogen receptor, *PR* progesterone receptor.

**Table 5:** ***APOA1 *rs670 A allele carrying patients had increased odds of post**-**surgery disease progression**

	**rs670**	**Unadjusted**		**Lymph node**-**adjusted**^**a**^		**ER/PR**-**adjusted**^**b**^	
		**OR (95% CI)**	***P *value**	**OR (95% CI)**	***P *value**	**OR (95% CI)**	***P *value**
Recurrence	G/G	1.00	-	1.00	-	1.00	-
	G/A	2.07 (1.03-4.14)	**0**.**041**	1.57 (0.74-3.33)	0.238	2.49 (1.14-5.44)	**0**.**022**
	A/A	3.44 (1.33-8.86)	**0**.**011**	2.83 (1.05-7.61)	0.040	3.08 (1.08-8.78)	**0**.**035**
Mortality	G/G	1.00	-	1.00	-	1.00	-
	G/A	1.82 (0.74-4.48)	0.191	2.25 (0.83-6.11)	0.112	1.05 (0.4-2.76)	0.925
	A/A	6.03 (2.08-17.51)	**0**.**001**	5.14 (1.56-16.97)	**0**.**007**	4.45 (1.4-14.15)	**0**.**010**

NOTE: Data shown represent number of patients (% of n) or mean±s.d.. Bold type indicates *p*<0.050.

^a^, adjusted for lymph node status, n=211.

^b^, adjusted for ER/PR status, n=196.

Abbreviations: *CI* confidence interval, *ER* estrogen receptor, *PR* progesterone receptor, *OR* odds ratio.

### *APOA1* rs670 A/A carriers had the worst outcome in lymph node-negative patients

To compare *APOA1* rs670 A/A carriage with the other known risk factors in post-surgery prognosis, we performed Cox regression analysis including ER/PR status, lymph node involvement, age, BMI and post-surgery adjuvant in adjusted models. Of specific importance, lymph node-positivity, ER/PR-negativity, combined therapy, and *APOA1* rs670 A/A carriage showed increased recurrence and mortality risk in unadjusted models (Table 6). The increased risk of recurrence and mortality observed in *APOA1* rs670 A/A carriers remained significant after adjustments for lymph node involvement, ER/PR status, age, BMI, or post-surgery adjuvant (Additional file 4). When testing the prognostic effects of lymph node, ER/PR, post-surgery adjuvant and *APOA1* rs670 A/A carrying status in an adjusted model, the significance of lymph node and *APOA1* rs670 A/A was stronger than of ER/PR and post-surgery adjuvant (Table 6). Lymph node positivity showed increased risks of recurrence (HR=2.62, 95% CI=1.43-4.79, *p*= 0.002) and mortality (HR= 5.35, 95% CI=2.25-12.68, *p*< 0.001), while *APOA1* rs670 A/A carriage in recurrence (HR=3.12, 95% CI=1.29-7.56, *p*=0.012) and mortality (HR=4.36, 95% CI=1.52-12.47, *p*= 0.006). In contrast, ER/PR status was associated with mortality but no longer with recurrence in the adjusted model, while post-surgery adjuvant shows a reversed pattern (Table 6).

**Table 6:** ***APOA1 *rs670 A**/**A carriage predicts poor post**-**surgery survival in breast cancer after adjustments for lymph node and ER**/**PR status**

	**Unadjusted**		**Adjusted (n=186)**	
	**HR (95% CI)**	***P *value**	**HR (95% CI)**	***P *value**
Recurrence				
Lymph node positivity^a^	2.86 (1.65-4.96)	<**0**.**001**	2.20 (1.18-4.10)	**0**.**013**
ER/PR negativity^b^	1.90 (1.08-3.37)	**0**.**027**	1.53 (0.85-2.77)	0.156
Age	1.01 (0.98-1.04)	0.479	-	-
BMI^c^	1.00 (0.93-1.08)	1.000	-	-
Combined therapy^d^	2.64 (1.46-4.78)	**0**.**001**	2.03 (1.01-4.08)	**0**.**046**
*APOA1* rs670 A/A^e^	4.02 (2.22-9.96)	**0**.**001**	3.02 (1.25-7.29)	**0**.**014**
Mortality				
Lymph node positivity^a^	6.01 (2.62-13.80)	<**0**.**001**	2.09 (1.01-4.29)	**0**.**046**
ER/PR negativity^b^	2.30 (1.13-4.70)	**0**.**022**	4.54 (1.88-10.94)	**0**.**001**
Age	1.03 (0.99-1.06)	0.127	-	-
BMI^c^	1.00 (0.91-1.10)	0.942	-	-
Combined therapy^d^	3.45 (1.50-7.98)	**0**.**004**	2.11 (0.84-5.31)	0.114
*APOA1* rs670 A/A^e^	6.30 (2.47-16.08)	<**0**.**001**	4.47 (1.56-12.79)	**0**.**005**

NOTE: *P* values were results of Cox proportional hazard regression analysis. Bold type indicates p<0.050.

^a^, compared with negative lymph node involvement, n=211.

^b^, compared with ER/PR positive, n=196.

^c^, n=185.

^d^, compared with TAM only.

^e^, compared with *APOA1* rs670 G/G.

Abbreviations: *BMI* Body mass index, *ER* estrogen receptor, *HR* Hazard ratio, *PR* progesterone receptor.

**Figure 1:**
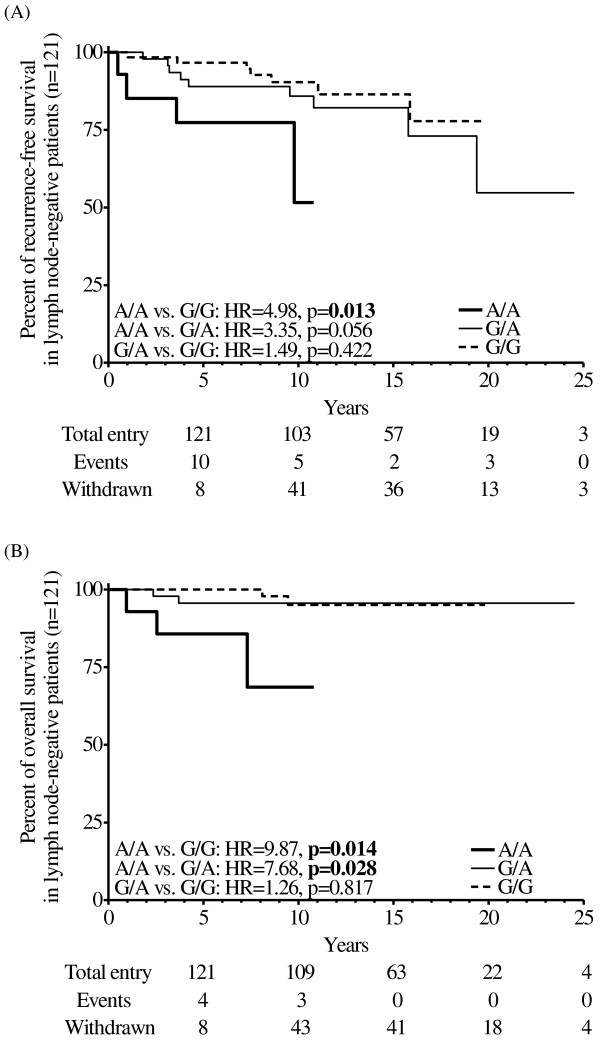
**Kaplan-Meier plots of breast cancer patient post**-**surgery survival stratified by *APOA1 *rs670 genotype.** The recurrence-free **(A)** and overall disease-specific survival **(B)** of *APOA1* A/A (bold line), G/A (thin line) and G/G (broken line) carrying lymph-node negative breast cancer patients were compared. The life tables are shown below the Kaplan-Meier survival plots. Bold type indicates *p*<0.050.

Therefore, to specifically rule out confounding from baseline lymph node involvement in predicting prognosis, we tested the prognostic effect of *APOA1* rs670 A/A carriage in baseline lymph node status-stratified patient groups. The prognostic effect of *APOA1* rs670 A/A carriage was only observed in the lymph node-negative patients (n=121, Table 7 and Figure 1) with higher hazard risks than non-stratified group (n=223, Table 4 and Additional file 5 upper panels). The prognostic effect of *APOA1* rs670 A/A carriage did not reach statistical significance in node-positive patients (n=90, Table 7 and Additional file 5 lower panels). The risk of recurrence associated with *APOA1* rs670 A/A carriage was higher as compared with G/G (HR=4.98, 95% CI=1.40-17.70, *p*=0.013) in lymph node-negative breast cancer patients. The risk of mortality in lymph node-negative *APOA1* rs670 A/A carrying breast cancer patients was also higher than *APOA1* rs670 G/G counterparts (HR=9.87, 95% CI=1.60-60.81, *p*=0.014). The contributions from ER/PR status, age, BMI, or post-surgery adjuvant in predicting outcome remained insignificant in lymph node-status stratified groups (Table 7).

**Table 7:** ***APOA1 *rs670 A**/**A carriers has the worst post**-**surgery outcomes in lymph node**-**negative patients**

	**Cox proportional hazard regression**					
	**Lymph node negative (n=121)**			**Lymph node positive (n=90)**		
	**HR**	**95% CI**	***P *value**	**HR**	**95% CI**	***P *value**
Recurrence						
ER/PR negativity^a^	2.06	0.80-5.31	0.136	0.62	0.30-0.62	0.202
Age	1.01	0.96-1.05	0.788	1.01	0.98-1.01	0.582
BMI	0.95	0.84-1.08	0.450	1.02	0.92-1.02	0.703
Combined therapy^b^	2.02	0.83-4.93	0.120	2.12	0.85-5.29	0.107
*APOA1* rs670 A/A^c^	4.98	1.40-17.70	**0**.**013**	2.46	0.86-2.46	0.093
Mortality						
ER/PR negativity^a^	0.24	0.05-1.08	0.064	0.52	0.22-1.18	0.118
Age	1.05	0.98-1.13	0.196	1.02	0.99-1.06	0.262
BMI	0.89	0.71-1.11	0.296	1.03	0.91-1.16	0.648
Combined therapy^b^	1.83	0.41-8.18	0.430	2.65	0.89-7.88	0.081
*APOA1* rs670 A/A^c^	9.87	1.60-60.81	**0**.**014**	2.86	0.96-8.55	0.060

NOTE: Bold type indicates *p*<0.050.

^a^, compared with ER/PR positive.

^b^, compared with TAM only.

^c^, compared with *APOA1* rs670 G/G.

Abbreviations: *BMI* Body mass index, *ER* estrogen receptor, *HR* Hazard ratio, *PR* progesterone receptor.

## Discussion

Dyslipidemia is a known complication of TAM-treatment in breast cancer patients, and increases risk of coronary-artery diseases in patients [33]. Of equal importance, dyslipidemia has been observed in breast cancer patients prior to treatment, and this correlation is supported by epidemiological studies [8-11]. Concomitantly, usage of lipid-lowering drugs such as statin and niacin has been shown to associate with decreased breast cancer recurrence and risk [15-17]. SNPs on the *APOA1*/*C3*/*A5* gene cluster, which is involved in lipid metabolism, are highly associated with dyslipidemia, metabolic syndrome, insulin resistance and responsiveness to relative treatments [19]. However, the contribution of SNPs on the *APOA1*/*C3*/*A5* gene cluster to breast cancer is yet to be defined. In this study, we tested the effects of dyslipidemia-associated SNPs on the *APOA1*/*C3*/*A5* gene cluster: *APOA1* rs670, *APOC3* rs2854116, *APOC3* rs2854117, *APOA5* rs662799 and *APOA5* rs2075291 on breast cancer progression in a Taiwanese patient group with mainly operable early-stage tumors, and a 10-year follow-up interval. We showed that only *APOA1* rs670 out of the 5 tested SNPs was correlated to breast cancer, lymph node-positivity, ER/PR double-negativity at baseline. Furthermore, carriers of both minor alleles on *APOA1* rs670 had shortest survival time and highest risk in disease progression independent of baseline characteristics. Moreover, the prognostic value of *APOA1* rs670 A/A carriage in the worse post-surgery outcomes was most evident in lymph node-negative patients.

The rs670 SNP contains a G-to-A substitution at 75bp upstream of *APOA1* transcriptional start site, and is within a MSPI restriction enzyme recognition [19]. A cross-sectional study focusing on the association of *APOA1* SNPs with breast cancer and patient phenotype reported that the -75G/A polymorphism correlated with breast cancer risk at baseline [34]. However, the slight discrepancy in associations of -75G/A polymorphism with ER status observed by us and Hamrita et al. is likely due to ethnic (Taiwanese *vs*. Tunisian) and age differences (62.7% *vs*. 38.9% <50years) [34]. Nevertheless, in our longitudinal study we found that *APOA1* rs670 predicted worse outcome after adjustment for ER/PR status, indicating alternative contributions from *APOA1* rs670 in cancer progression. ApoA1 is the structural protein of HDL, and interacts with lecithin cholesterol acyl transferase, which controls the limiting steps in HDL maturation and therefore reverse cholesterol transport [19]. The minor allele of *APOA1* rs670 has been correlated to altered HDL-C, diabetes and coronary-artery disease: lower levels of HDL-C was observed in Northern Indians [20], and correlated with severe forms of cardiovascular diseases in Northern Indians, Caucasians in Spain and Australia [20,24,25]. Moreover, *APOA1* rs670 A/A Spanish carriers had a higher risk of diabetes than their non-A/A counterparts, though their HDL-C did not differ significantly [21,22]. As plasma apoA1 level is regulated by estrogen and thus TAM treatments, the timing of plasma apoA1 measurement in breast cancer patients is critical. However, we did not have access to TAM treatment-naïve plasma of the patients recruited in this study. Nevertheless, the disease phenotype associated *APOA1* rs670 remains significant without HDL-C differences, as has been reported in a number of studies on metabolic/cardiovascular diseases [21,24,25].

The significance of plasma HDL-C levels in breast cancer has long been investigated and debated [35]. The current held view is that higher HDL-C levels, which are preventive for cardiovascular events, are also protective for breast cancer. The majority of reports supporting this view were of case–control design, and found lower total or lipid-rich HDL, or higher total cholesterol/HDL-C ratio in breast cancer patients as compared with non-cancerous or other cancer controls in various ethnic groups [8,10,11]. A 17.2-year prospective cohort study carried out in Norway found that naive females with the lowest quartile of HDL-C had highest risk of later developing breast cancer [14]. Moreover, the same Norwegian group found the breast cancer patients with highest total cholesterol or lowest plasma HDL-C levels had highest risk in mortality [13]. The female breast is an active site of both lipid uptake and lipid secretion during periods of milk secretion, thus it is readily acceptable that apoA1 is present in human milk [36]. Supportively, apoA1 was also identified in the breast cancer tissues and *ex vivo* cultured medium. ApoA1 was found in breast tumors, and its amount was correlated positively with chemotherapy resistance in malignant tumors [37]. In contrast, breast tumors later found responsive to chemotherapy secreted higher amount of apoA1 than tumors non-responsive to chemotherapy during short term ex vivo culture, while non-cancerous tissues secreted highest amount of apoA1 [38]. Furthermore, a successful reduction of breast cancer growth in mice by vitamin D treatment was accompanied by decrease of apoA1 production in tumor tissues [39]. It is at this stage unknown how much apoA1 in circulation is contributed by the mammary tissue, but the retention of apoA1 in breast cancer tissues is in accordance with the observation of lower plasma HDL/apoA1 in breast cancer patients. Though the association of plasma lipid profiles and breast cancer incidence or progression is strongly and continuously suggested, the molecular mechanisms of HDL or apoA1 in promoting breast cancer risk are still unclear.

Plasma apoA1/lipid-poor HDL bind to ATP-binding cassette (ABC) lipid transporters during maturation, and the intracellular cholesterol is exported to load onto HDL, facilitating reverse cholesterol transport [40]. Several members of the ABC family were found in mammary tissues, but only those associated with drug resistance were increased in breast cancers as compared with normal tissues [41]. In contrast, ABC transporters involved in lipid transport, including ABC-A1 and ABC-A3, were decreased in breast cancer tissues as compared with healthy mammary glands [42]. *In vitro* studies found that over-expression of ABC-G1 in breast cancer cell line induces cellular cholesterol efflux and apoptosis [43]. Moreover, ABCA- or ABCG-HDL binding concomitantly induces intracellular signaling and suppresses inflammatory reactions, which is known to be a key factor in tumorigenesis. Binding of ABCA or ABCG by apoA1 induces the recruitment of phosphrylated STAT-3 and JAK2, and thus suppresses the inflammatory pathways including TL4-signalling [40]. Furthermore, ABCA induces Cdc42 and PAK-1 signaling, which in turn are regulative for breast cancer ER/PR expression *in vitro* and *in vivo*[44,45]. Therefore, the altered HDL/apoA1 levels observed in *APOA1* rs670 A carriers may participate in the tumorigenesis, survival, ER/PR status of breast cancer cells. In a previous published study, we found that genetic and intra-tumoral ER polymorphisms were correlated with breast cancer in Taiwanese females [30,31]. It would be of interest how *APOA1* rs670 and HDL-C interact with ER polymorphisms both at a systemic and local (mammary tissue) level in these patients.

Clinically, our finding of worst outcome in *APOA1* rs670 A/A breast cancer patients as compared to their non-A/A counterparts provides pivotal implications in personalized treatment regimen and therapeutic strategies. The median recurrence-free and overall survival years of *APOA1* rs670 A/A carrying breast cancer patients were both longer than the standard chemotherapy duration (~1 year) and the advised 5-year limit for continuous TAM usage. As the patients recruited in this study were non-obese and of early tumor-staging, the administration of adjuvant therapy may be less intensive. Furthermore, the prognostic value of *APOA1* rs670 was even more evident in the lymph node-negative patients, who were given less adjuvant therapies under the current assessment guidelines. Thus *APOA1* rs670 A/A carriers may benefit from advanced therapies. Recent studies demonstrated that administration of apoA1 mimetic peptides reduced the development of cancer in murine models [46,47]. Furthermore, a large cohort study on the effect of post-diagnosis statin use in breast cancer females reported a lowered risk in cancer recurrence [48]. These reports suggest that raising HDL-C or apoA1 may be preventive or therapeutic for breast cancers, especially in the *APOA1* rs670 A/A carrying patients. On the other hand, due to the low plasma HDL-C and tumor ER/PR negativity in the *APOA1* rs670 A/A carriers, the usage of TAM in these breast cancer patients may be controversial and require further evaluation.

## Conclusions

In conclusion, we demonstrated the correlation of dyslipidemia-associated *APOA1* rs670 minor allele with unfavorable baseline characteristics in Taiwanese breast cancer patients, and the 10-year follow-up revealed patients carrying both minor alleles had worst survival in lymph node-negative patients. Though TAM treatment naïve plasma HDL-C levels of the recruited patients were unavailable, our data was consistent after appropriate adjustments for possible confounding factors. Our study provides novel clinical directions for both personalized treatment regimen and therapeutic strategies.

## Abbreviations

HDL-C: High density lipoprotein-cholesterol; Apo: Apolipoproteins; SNP: Single-nucleotide polymorphisms; BMI: Body mass index; ER: Estrogen receptor; PR: Progesteron receptor; TAM: Tamoxifen.

## Competing interests

The authors declare that they have no competing interests.

## Author contributions

MCH, WCH, and KCY conceived and designed the study. MCH and KCY developed the methodology, performed laboratory experiments, and supervised the study. MCH, WCH, and KTL acquired and prepared clinical data. MCH, WCH, KTL, CHW, HYS, ILL and KCY analyzed and interpreted the data. MCH, WCH, KTL, CHW, HYS, ILL and KCY wrote, reviewed and edited the manuscript. All authors read and approved the final manuscript.

## Pre-publication history

The pre-publication history for this paper can be accessed here:

http://www.biomedcentral.com/1471-2407/13/330/prepub

## Supplementary Material

Additional file 1 **The distributions of tested SNPs on *APOA1*/*C3*/*A5 *gene cluster in breast cancer patients and healthy controls.** Click here for file

Additional file 2 **The linkage disequilibrium pattern of tested *APOA1*/*C3*/*A5 *SNP gene cluster in the study group.** The SNP linkage pattern in healthy controls (**A**) and breast cancer patients (**B**) are shown. The upper panels show the relative chromosomal localization of the five SNPs, and the lower panels the test results of linkage disequilibrium by Haploview. The colour scale shown in the lower panels demonstrates high linkage disequilibrium (red) to minimal linkage disequilibrium (white). The numbers in the lower panels represent the pair-wise D’ values which are shown in two digits after the decimal point. Linkage tests with LOD values ≥2 and D’ values >0.80 were considered as presence of significant linkage. D’ values of 1.00 are not shown. Click here for file

Additional file 3 **The allele frequencies of tested *APOA1*/*C3*/*A5 *SNPs in breast cancer patients and healthy controls.** Click here for file

Additional file 4 **The comparison of *APOA1 *rs670 A/A carriage with other risk factors in predicting disease progression.** Click here for file

Additional file 5 **The Kaplan-Meier survival plots of *APOA1 *rs670 genotype carrying breast cancer patients.** The recurrence-free (**A** and **C**) and overall disease-specific survival (**B** and **D**) of *APOA1* A/A (bold line), G/A (thin line) and G/G (broke line) carrying breast cancer patients were compared. Data from all recruited patients (n=223) are shown in A & B, and lymph-node positive patients (n=90) in **C** &**D**. The life tables are shown below the Kaplan-Meier plots. Click here for file
