# Supplementary material for: The dyslipidemia-associated SNP on the APOA1/C3/A5 gene cluster predicts post-surgery poor outcome in Taiwanese breast cancer patients: a 10-year follow-up study
Source: BMC Cancer. 2013 Jul 5;13:330. doi: 10.1186/1471-2407-13-330 (PMC3708770; doi:10.1186/1471-2407-13-330)
Supplement: Additional file 1 — The distributions of tested SNPs on APOA1/C3/A5 gene cluster in breast cancer patients and healthy controls. [file 1471-2407-13-330-S1.doc]

Additional file 1. The distributions of tested SNPs on *APOA1/C3/A5* gene cluster in breast cancer patients and healthy controls.

| SNP | Genotype | Breast cancer patients (n=223) | |  | Healthy controls  (n=162) | |  | *P* value | | |
| --- | --- | --- | --- | --- | --- | --- | --- | --- | --- | --- |
|  |  | n | % of n |  | n | % of n |  | G/G *vs.* G/A *vs.* A/A | G/G *vs.*  (G/A + A/A) | (G/G + G/A) *vs.* A/A |
| *APOA1* rs670 | G/G | 90 | 40.36 |  | 83 | 51.23 |  | 0.106 | **0.038** | 0.517 |
|  | G/A | 106 | 47.53 |  | 63 | 38.89 |  |  |  |  |
|  | A/A | 27 | 12.11 |  | 16 | 9.88 |  |  |  |  |
|  | HWE *p*-value | 0.755 |  |  | 0.520 |  |  |  |  |  |
|  |  |  |  |  |  |  |  |  |  |  |
| *APOC3* rs2854116 | T/T | 75 | 33.63 |  | 48 | 29.63 |  | 0.559 | 0.578 | 0.303 |
|  | T/C | 107 | 47.98 |  | 78 | 48.15 |  |  |  |  |
|  | C/C | 41 | 18.39 |  | 36 | 22.22 |  |  |  |  |
|  | HWE *p*-value | 0.874 |  |  | 0.775 |  |  |  |  |  |
|  |  |  |  |  |  |  |  |  |  |  |
| *APOC3* rs2854117 | C/C | 69 | 30.94 |  | 44 | 27.16 |  | 0.341 | 0.430 | 0.192 |
|  | T/C | 116 | 52.02 |  | 81 | 50.00 |  |  |  |  |
|  | T/T | 38 | 17.04 |  | 37 | 22.84 |  |  |  |  |
|  | HWE *p*-value | 0.460 |  |  | 1.000 |  |  |  |  |  |
|  |  |  |  |  |  |  |  |  |  |  |
| *APOA5* rs662799 | T/T | 108 | 48.43 |  | 84 | 51.85 |  | 0.149 | 0.536 | 0.055 |
|  | T/C | 88 | 39.46 |  | 68 | 41.98 |  |  |  |  |
|  | C/C | 27 | 12.11 |  | 10 | 6.17 |  |  |  |  |
|  | HWE *p*-value | 0.217 |  |  | 0.598 |  |  |  |  |  |
|  |  |  |  |  |  |  |  |  |  |  |
| *APOA5* rs2075291 | G/G | 198 | 88.79 |  | 139 | 85.80 |  | 0.634 | 0.435 | 1.000 |
|  | G/T | 22 | 9.87 |  | 21 | 12.96 |  |  |  |  |
|  | T/T | 3 | 1.35 |  | 2 | 1.23 |  |  |  |  |
|  | HWE *p*-value | 0.087 |  |  | 0.469 |  |  |  |  |  |

NOTE: *P*-values were results of Chi-squared analysis. Bold type indicates *p*<0.050.

Abbreviations: HWE, Hardy-Weinberg equilibrium.
