# Supplementary figures and images for: The dyslipidemia-associated SNP on the APOA1/C3/A5 gene cluster predicts post-surgery poor outcome in Taiwanese breast cancer patients: a 10-year follow-up study
Source: BMC Cancer. 2013 Jul 5;13:330. doi: 10.1186/1471-2407-13-330 (PMC3708770; doi:10.1186/1471-2407-13-330)

Additional file 2.

(A) (B)


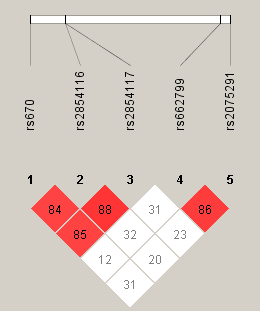

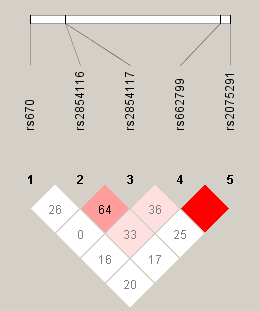

Supplement: Additional file 2 — The linkage disequilibrium pattern of tested APOA1/C3/A5 SNP gene cluster in the study group. The SNP linkage pattern in healthy controls (A) and breast cancer patients (B) are shown. The upper panels show the relative chromosomal localization of the five SNPs, and the lower panels the test results of linkage disequilibrium by Haploview. The colour scale shown in the lower panels demonstrates high linkage disequilibrium (red) to minimal linkage disequilibrium (white). The numbers in the lower panels represent the pair-wise D’ values which are shown in two digits after the decimal point. Linkage tests with LOD values ≥2 and D’ values >0.80 were considered as presence of significant linkage. D’ values of 1.00 are not shown. [file 1471-2407-13-330-S2.doc]
