# Supplementary material for: The dyslipidemia-associated SNP on the APOA1/C3/A5 gene cluster predicts post-surgery poor outcome in Taiwanese breast cancer patients: a 10-year follow-up study
Source: BMC Cancer. 2013 Jul 5;13:330. doi: 10.1186/1471-2407-13-330 (PMC3708770; doi:10.1186/1471-2407-13-330)
Supplement: Additional file 3 — The allele frequencies of tested APOA1/C3/A5 SNPs in breast cancer patients and healthy controls. [file 1471-2407-13-330-S3.doc]

Additional file 3. The allele frequencies of tested *APOA1/C3/A5* SNPs in breast cancer patients and healthy controls.

NOTE: *P-*values were results of Chi-squared analysis.

| SNP | Allele | Allele frequency (%) | | *P* value |
| --- | --- | --- | --- | --- |
|  |  | Breast cancer | Healthy control |  |
| rs670 | G | 64.13 | 70.68 | 0.056 |
|  | A | 35.87 | 29.32 |  |
|  |  |  |  |  |
| rs2854116 | T | 57.62 | 53.70 | 0.279 |
|  | C | 42.38 | 46.30 |  |
|  |  |  |  |  |
| rs2854117 | C | 56.95 | 52.16 | 0.187 |
|  | T | 43.05 | 47.84 |  |
|  |  |  |  |  |
| rs662799 | T | 68.16 | 72.84 | 0.161 |
|  | C | 31.84 | 27.16 |  |
|  |  |  |  |  |
| rs2075291 | G | 93.72 | 92.28 | 0.436 |
|  | T | 6.28 | 7.72 |  |
