# Supplementary material for: The dyslipidemia-associated SNP on the APOA1/C3/A5 gene cluster predicts post-surgery poor outcome in Taiwanese breast cancer patients: a 10-year follow-up study
Source: BMC Cancer. 2013 Jul 5;13:330. doi: 10.1186/1471-2407-13-330 (PMC3708770; doi:10.1186/1471-2407-13-330)
Supplement: Additional file 4 — The comparison of APOA1 rs670 A/A carriage with other risk factors in predicting disease progression. [file 1471-2407-13-330-S4.doc]

Additional file 4. The comparison of *APOA1* rs670 A/A carriage with other risk factors in predicting disease progression.

|  | Lymph node status adjusted (n=211) | |  | ER/PR status adjusted (n=196) | |  | Age adjusted  (n=223) | |  | BMI adjusted  (n=185) | |  | Combined therapy adjusted (n=213) | |
| --- | --- | --- | --- | --- | --- | --- | --- | --- | --- | --- | --- | --- | --- | --- |
|  | HR  (95% CI) | p value |  | HR  (95% CI) | p value |  | HR  (95% CI) | p value |  | HR  (95% CI) | p value |  | HR  (95% CI) | p value |
| Recurrence |  |  |  |  |  |  |  |  |  |  |  |  |  |  |
| Lymph node  involvement | 2.64  (1.49-4.65) | **0.001** |  | - | - |  | - | - |  | - | - |  | - | - |
| ER/PR negative | - | - |  | 1.61 (0.90-2.88) | 0.111 |  | - | - |  | - | - |  | - | - |
| Age | - | - |  | - | - |  | 1.01  (0.98-1.04) | 0.551 |  | - | - |  | - | - |
| BMI | - | - |  | - | - |  | - | - |  | 0.99  (0.92-1.07) | 0.875 |  | - | - |
| Combined therapy | - | - |  | - | - |  | - | - |  | - | - |  | 3.66 (1.64-8.18) | **0.003** |
| *APOA1* rs670 A/A^a^ | 3.22 (1.44-7.20) | **0.004** |  | 3.83 (1.59-9.23) | **0.003** |  | 4.01  (1.83-8.80) | **0.001** |  | 3.96  (1.72-9.08) | **0.001** |  | 2.51 (1.38-4.57) | **0.002** |
|  |  |  |  |  |  |  |  |  |  |  |  |  |  |  |
| Death |  |  |  |  |  |  |  |  |  |  |  |  |  |  |
| Lymph node  involvement | 5.77 (2.46-13.54) | **<0.001** |  | - | - |  | - | - |  | - | - |  | - | - |
| ER/PR negative | - | - |  | 1.85 (0.89-3.84) | 0.098 |  | - | - |  | - | - |  | - | - |
| Age | - | - |  | - | - |  | 1.03  (0.99-1.06) | 0.134 |  | - | - |  | - | - |
| BMI | - | - |  | - | - |  | - | - |  | 0.99  (0.89-1.09) | 0.758 |  | - | - |
| Combined therapy | - | - |  | - | - |  | - | - |  | - | - |  | 5.96 (2.24-15.88) | <0.001 |
| *APOA1* rs670 A/A^a^ | 4.01 (1.56-10.33) | **0.004** |  | 5.68 (1.97-16.38) | **0.001** |  | 6.18  (2.43-15.77) | **<0.001** |  | 6.45  (2.32-17.95) | **<0.001** |  | 3.25 (1.4-7.54) | **0.006** |

NOTE: ^a^, compared with *APOA1* rs670 G/G. *P*-values were results of Cox regression analysis. Bold type indicates p<0.050.

Abbreviations: BMI, Body mass index; ER, estrogen receptor; HR, Hazard ratio; PR, progesterone receptor.
