# Supplementary material for: The dyslipidemia-associated SNP on the APOA1/C3/A5 gene cluster predicts post-surgery poor outcome in Taiwanese breast cancer patients: a 10-year follow-up study
Source: BMC Cancer. 2013 Jul 5;13:330. doi: 10.1186/1471-2407-13-330 (PMC3708770; doi:10.1186/1471-2407-13-330)
Supplement: Additional file 5 — The Kaplan-Meier survival plots of APOA1 rs670 genotype carrying breast cancer patients. The recurrence-free (A and C) and overall disease-specific survival (B and D) of APOA1 A/A (bold line), G/A (thin line) and G/G (broke line) carrying breast cancer patients were compared. Data from all recruited patients (n=223) are shown in A & B, and lymph-node positive patients (n=90) in C &D. The life tables are shown below the Kaplan-Meier plots. [file 1471-2407-13-330-S5.doc]

Additional file 5.
